# Prevalence and Associations of Hypertension in Sri Lankan Adults: Estimates from the SLHAS 2018–19 Survey Using JNC7 and ACC/AHA 2017 Guidelines

**DOI:** 10.5334/gh.1135

**Published:** 2022-08-01

**Authors:** Ravindra P. Rannan-Eliya, Nilmini Wijemunige, Prasadini Perera, Yasodhara Kapuge, Nishani Gunawardana, Chathurani Sigera, H. M. M. Herath, Bilesha Perera, Anuji Gamage, Nethmi Weerawardena, Ishwari Sivagnanam, SLHAS Collaborators

**Affiliations:** 1 Institute for Health Policy (IHP), LK; 2 University of Peradeniya, LK; 3 University of Colombo, LK; 4 University of Ruhuna, LK; 5 General Sir John Kotelawala Defence University, LK; 6 SLHAS Collaborators—Vajira H. W. Dissanayake (University of Colombo), Shanti Dalpatadu (IHP), Sarath Samarage (IHP), Upeka Samarakoon (IHP), U. M. N. P. Samaranayake (Ministry of Health), and Cheroni Pullenayegam (IHP), LK

**Keywords:** Hypertension, blood pressure, prevalence, design effects

## Abstract

**Background:**

Sri Lanka lacks robust estimates of hypertension (HTN) prevalence owing to few national studies, hindering optimization of control strategies. Evidence on how the revised 2017 American College of Cardiology/American Heart Association (ACC/AHA) HTN definition affects prevalence in low- and middle-income countries (LMICs) is also limited.

**Objectives:**

To make robust estimates of HTN prevalence in the Sri Lankan adult population, and to assess impact of the ACC/AHA 2017 definitions.

**Methods:**

Data were sourced from the 2018–2019 first wave of the Sri Lanka Health and Ageing Study (SLHAS), a nationally representative longitudinal study of the noninstitutionalized adult population. After excluding those with missing data and aged <18 years, 6,342 participants (95.1%) were included in the analysis. HTN was defined using either the traditional threshold of systolic BP (SBP) ≥140 mmHg or a diastolic BP (DBP) ≥90 mmHg, or the ACC/AHA 2017 threshold of SBP ≥130 mmHg or DBP ≥80 mmHg, or if taking antihypertensive medication.

**Results:**

Estimated prevalence of HTN in all Sri Lankan adults was 28.2% using the traditional definition, and it doubled to 51.3% when applying the ACC/AHA 2017 definition. Of those classified as hypertensive according to the older and ACC/AHA 2017 definitions, 53.4% and 31.2%, respectively, were previously diagnosed. Of the 23.2% of adults reclassified as hypertensive by the ACC/AHA 2017 definition, 16.6% had a history of CVD or diabetes. Increased prevalence was associated with urban residence, socioeconomic status, obesity, and Muslim ethnicity. Prevalence increased with age, but the increase was steeper in women from their 30s.

**Conclusions:**

Nearly one in three adult Sri Lankans are hypertensive, requiring antihypertensive treatment. Applying the ACC/AHA 2017 definitions almost doubles numbers, but many of those reclassified would require treatment under recent WHO guidelines. Study findings also suggest that design effects in HTN surveys may be higher than usually assumed.

## Introduction

Hypertension (HTN) is a leading risk factor for cardiovascular disease (CVD) and chronic kidney disease (CKD), and prevalence is increasing in low- and middle-income countries (LMICs) [1]. The 2030 Sustainable Development Goals set specific targets to lower blood pressure and increase treatment coverage, reflecting global commitments to reverse this trend. To track progress and plan interventions, countries need robust information on levels and trends.

HTN—the raised blood pressure (BP) levels at which intervention is usually indicated—is traditionally defined using a threshold of a systolic BP (SBP) ≥140 mmHg or a diastolic BP (DBP) ≥90 mmHg, as described by the 2013 guidelines of the European Society of Hypertension/European Society of Cardiology (ESH/ESC) and the Seventh Report of the Joint National Committee on Prevention, Detection, Evaluation, and Treatment of High Blood Pressure (JNC7) [2,3]. This threshold involves a trade-off between the costs and benefits of expanded treatment, as the influence of BP on CVD and CKD is log-linear and extends below this threshold, and therapy provides similar proportional risk reductions irrespective of pretreatment blood pressure [4]. In 2017, the American College of Cardiology/American Heart Association (ACC/AHA) issued new guidelines [5] that lowered the SBP and DBP diagnostic thresholds to 130 mmHg and 80 mmHg, respectively, and recommended medication for those with Stage 1 HTN (defined as SBP 130–139 mmHg or DBP 80–89 mmHg) who had preexisting CVD or high CVD risk. Although the 2021 World Health Organization (WHO) hypertension treatment guidelines and expert review did not similarly redefine hypertension, the practical difference is less as these also recommend medication for individuals with SBP 130–139 mmHg who have diabetes, CVD, or high CVD risk [6]. Nevertheless, a key consideration for whether the ACC/AHA 2017 redefinition should be adopted globally is how many people would be affected. Only a few studies in LMICs, such as China, Iran, and Ghana, have investigated this, and these suggest that applying the ACH/AHA guidelines could increase HTN prevalence by 58–220% [7,8,9].

Sri Lanka is a LMIC located in South Asia that is experiencing rapid transformations associated with increased risk of hypertension, including aging, increased affluence, physical inactivity, urbanization, and high levels of salt intake [10,11]. Despite this, information on HTN prevalence, trends, and patterns is limited. Reasons include few population surveys, variations in study definitions, and lack of detailed results or data access for completed studies. Since 1985, only nine population-based surveys of hypertension have been published, most covering only subnational areas [12,13,14,15,16,17,18,19,20]. One WHO STEPS study in 2015 was national, reporting a prevalence of 26.1%, but this excluded adults aged >69 years and did not release estimates of prevalence in subgroups [18].

This study exploits a national survey that covered all ages to assess the prevalence of HTN in the full adult population of Sri Lanka and by major characteristics, using both the older JNC7 and newer ACC/AHA 2017 definitions.

## Methods

### Study design and participants

We use data from the first 2018–2019 wave of the Sri Lanka Health and Ageing Study (SLHAS). The SLHAS is a national, longitudinal cohort study managed by a consortium of the Institute for Health Policy, University of Colombo, University of Peradeniya, University of Ruhuna, and the University of Rajarata, and it is approved by the Ministry of Health (MOH), Sri Lanka.

The SLHAS used a stratified, multistage probability design to recruit a nationally representative sample of the noninstitutionalized adult population of Sri Lanka. Stratification involved two steps. In the first, following the conventional approach in Sri Lanka, all grama niladhari divisions (GNDs) (*N* = 14,104), which are the smallest administrative unit in Sri Lanka and primary sampling units (PSUs) in the SLHAS, were categorized by district (*N* = 25) and sector of residence (urban, rural, estate, rural/estate) into 57 preliminary strata. In a second innovative step, PSUs (GNDs) within each preliminary stratum were substratified into equally sized population quantiles after having ranked them using an index of area socioeconomic status (SES), generating 157 strata. This index was constructed by applying principal components analysis (PCA) to a set of GND-level indicators derived from the 2012 national population census obtained from the Department of Census and Statistics (DCS), such as the percentages of adults in different employment categories or the percentages of households using different cooking fuels, supplemented by a GND-level poverty index estimated by DCS using unpublished 2012 census data [21]. This was expected to reduce survey design effects, as many health indicators would be correlated with the GND SES index which would make stratification beneficial [22], and given previous research showing that SES area stratification performs better than traditional area stratification [23].

Within strata, individuals were sampled using multistage, probability cluster sampling. In the first stage, a minimum of two PSUs were selected from each stratum using probability proportionate to the size of their adult population, with disproportionately large PSUs picked with certainty as an implicit additional layer of stratification. At the second stage, four to six widely spaced households were identified in each PSU by systematically sampling the electoral register, or in rural areas where households were not distributed by street by using satellite maps showing the distribution of buildings to pick dispersed geolocations. Recruitment teams visited these households or the household nearest to the sampled geolocation, and then additional households at preset interhousehold intervals of two to four by walking in a predefined track, with larger intervals in more densely populated or urban PSUs. If they gained entry, recruiters enumerated all household residents and recorded data about the household and its residents, using a computer-assisted personal interviewing (CAPI) application running on iPad computer tablets. If the household gave consent, the CAPI software randomly selected one adult (≥18 years) using weighted probabilities that targeted a final equal distribution of respondents by sex and by age up to 69 years with oversampling of those aged ≥70 years. The study protocol excluded pregnant women and adults unable to give informed consent, and if the selected individual declined participation, the whole household was excluded, with the CAPI system preventing recruiters from selecting another individual.

Selected individuals were invited to attend a field clinic near their residence, typically a MOH clinic, where they were interviewed to assess their health and collect other individual and household information, and they underwent a medical examination and collection of biomarkers, including anthropometric measures and blood pressure. Individuals with mobility limitations were interviewed at home with a shorter examination that included blood pressure.

The Sri Lanka Medical Association Ethical Review Committee (ERC/18-022) approved the study. Study information was provided to all participants, together with an official letter from MOH encouraging participation, and all participants gave informed written consent.

### Survey completion

Data collection took place from November 9, 2018 to November 14, 2019, with field work staggered within districts to minimize seasonal bias. Out of 10,689 sampled households, recruiters contacted 10,247 (95.9%), of which 185 (1.8%) refused participation. Of the 10,062 individuals who agreed to participate, 6,627 were interviewed at a field clinic and 41 completed home interviews, giving an effective response rate of 65.0%. Response rates were higher in women (69%), known diabetics (73%), adults ≥45 years (74%), and rural areas (70%), and they were lower in Muslim women. Feedback by field recruiters indicate that younger adults were less likely to see the study as beneficial or relevant, whereas Muslim women in some communities were reluctant or discouraged for sociocultural reasons to attend field clinics alone or without spousal permission. The terrorist attacks in Sri Lanka in April 2019 caused a six-week disruption of field work leading to noncoverage of several PSUs, a fall in Muslim response rates, and the inability to survey one predominantly Muslim PSU owing to security conditions. The study team replaced the affected PSU with a substitute PSU from the same stratum, but it proved impossible to match the ethnic profile of the original PSU. Other PSUs that could not be covered were handled by merging 40 strata that were left with single PSUs with adjacent strata to yield 117 final strata.

### Blood pressure measurement

All field clinic staff, who were medical or nursing graduates, were trained on the use of the study’s BP measurement protocol, including preparation of participants and using the SLHAS CAPI platform running on iPads. BP was measured after the participant rested for 10 minutes in a sitting position and with the arm resting at the level of the heart. All measurements were taken using the right arm unless specific conditions prohibited this. Two readings were obtained, 10 minutes apart, using an OMRON HEM-7320 Automatic BP Monitor, with a preformed flexible cuff allowing for arm circumferences of 17–36 cm. Accuracy of all devices was validated against a mercury column sphygmomanometer.

### Other data collection

The participant interview, which also checked personal medical records and medicines that the participant brought with them, assessed whether the individual had evidence of prior diagnoses of HTN, diabetes mellitus, and other chronic conditions; symptoms consistent with angina or intermittent claudication (ROSE criteria) [24]; history of acute myocardial infarction (AMI) or stroke; and recorded medicines used in the previous two weeks. Individuals with medical records, symptoms, or self-reported history consistent with angina, intermittent claudication, AMI, or stroke were classified as having history of cardiovascular or peripheral vascular disease (CVD/PVD). Medicines were coded to the WHO Anatomical Therapeutic Chemical (ATC) classification, with antihypertensive medications defined as medicines in ATC classes C03A, C07–C08, C09A–C09D, C10BX03–C10BX04, C10BX06–C10BX07, C10BX09–C10BX15, and C09C–C09D.

Height was measured using a Seca 240 cm height measure to the nearest 0.1 cm. Weight was measured using an OMRON BF511 Body Composition Monitor to the nearest 0.1 kg. BMI was calculated as weight (kg) divided by the square of height (m). Waist circumference measurement was collected from participants using a Seca 200 cm tape measure at the level of natural indent of trunk during expiration.

Information on participant household assets was used to generate a SES ranking through PCA and to group all respondents by SES quantiles.

### Hypertension definitions

Participants’ SBP and DBP were taken as the average of the two recordings. Hypertension (HTN) was defined in two ways. Following the JNC7 definition, individuals who had a SBP ≥140 mmHg or a DBP ≥90 mmHg or took any antihypertensive medications were categorized as hypertensive (HTN-JNC7). To define hypertension according to ACC/AHA 2017 definitions (HTN-ACC/AHA), a lower threshold of a systolic BP ≥130 mmHg or a diastolic BP ≥80 mmHg was used.

### Prevalence estimation

Owing to its complex sampling design, SLHAS data are weighted to make population-level inferences. For all participants, the SLHAS generates a nonresponse weight using multilevel logistic regression that models the propensity to participate as a function of individual, household, area, and operational characteristics recorded during recruitment, including sex, age, language, whether self-reporting as diabetic, household SES, and operational details such as day of week. Ethnicity or whether the respondent was a known hypertensive were not recorded at recruitment and were not used.

The original study design required these weights to be calibrated so their sums matched population totals at the level of each stratum, district, and province for each of 14 age-sex groups (age groups: 18–29, 30–39, 40–49, 50–59, 60–69, 70–79, 80+), with population estimates sourced from the 2012 national census with adjustment for demographic change to 2019. This would not address potential bias from the observed underrepresentation of Muslims, which the nonresponse weighting did not consider. Consequently, this was revised to include three calibration steps done iteratively at increasing levels of aggregation (stratum > district > province > national): (i) population totals (stratum > national); (ii) age-sex totals (district > national); and (iii) ethnic totals (province > national), with weight trimming at selected points. As ethnicity is diverse and often contested in Sri Lanka, we defined Muslim ethnicity as those who self-described themselves as “Muslim,” “Moor,” or “Malay” when asked about ethnicity.

We estimated prevalence using these final weights, accounting for the clustered sampling design with a finite population correction and estimating variances using Taylor linearization. For generating estimates for international comparison, a second set of weights, recalibrated to match the WHO standard population [25], were also compiled. All analyses were performed using statistical software Stata version 17.0.

### Evaluation of sample and weighting design

We evaluated several design features intended to improve precision and reduce bias, to inform future research. To assess area SES stratification, we examined the relationship with HTN using bivariate and categorical analysis, as well as localized regression. We evaluated the inclusion of ethnicity in the poststratification weighting by repeating the analysis without it. We examined the extent of spatial autocorrelation in HTN, which reduces precision when using cluster sampling, by using Stata’s loneway command to calculate intraclass correlation coefficients (ICCs), and we evaluated the overall study design by estimating design effects (DEFFs), which is simply the ratio of the actual sample size to the sample size that would yield the same precision when using simple random sampling.

## Results

### Characteristics of participants

We excluded 3 SLHAS participants aged <18 years and 323 with missing data for taking antihypertensive medication or lacking either SBP or DBP measurements, leaving 6,342 (95.1%) participants for analysis from 298 PSUs. Their mean age (±SD) was 49.9 (±17.2), and 51.0% (3,235) were female, 16.9% (1,070) were previously diagnosed diabetics, and 23.6% (1,495) were self-reported as hypertensive or were taking antihypertensive medication. The sample had underrepresentation of younger adults and Muslims but, after weighting, matched the national population on age, sex, ethnicity, and area and household SES (Table 1).

**Table 1:** Characteristics of Sri Lankan adults in the SLHAS 2018–19 hypertension sample (unweighted sample and weighted percentage).


SAMPLE CHARACTERISTICS	UNWEIGHTED *N*	UNWEIGHTED %/MEAN (SD)	WEIGHTED %/MEAN (SD)	REFERENCE VALUE

**Age (Mean)**	6,342	49.9 (17.2)	43.9 (16.7)	–

18–29	932	14.7	25.1	25.1

30–39	1,088	17.2	20.8	20.8

40–49	1,126	17.8	18.0	18.0

50–59	1,101	17.4	16.2	16.2

60–69	1,099	17.3	11.6	11.6

70–79	821	13.0	6.1	6.1

80+	175	2.8	2.1	2.1

**Sex**				

Male	3,107	49.0	47.6	47.6

Female	3,235	51.0	52.4	52.4

**Ethnicity**				

Sinhala	4,443	70.1	74.9	74.9

Tamil	1,464	23.1	15.3	15.3

Muslim	411	6.5	9.5	9.5

Other	24	0.6	0.3	0.3

**Education**				

No formal schooling	241	3.8	3.0	–

Primary education	871	13.8	10.0	–

Secondary education	4,959	78.3	82.4	–

Tertiary education	259	4.1	4.6	–

**Sector**				

Urban	1,923	30.3	22.6	–

Rural	3,483	54.9	66.8	–

Estate	168	2.6	0.7	–

Rural/estate	768	12.1	9.9	–

**Province**				

Western	1,338	21.1	30.0	–

Central	929	14.6	12.5	–

Southern	812	12.8	12.3	–

Northern	678	10.7	5.0	–

Eastern	534	8.4	6.8	–

North-Western	515	8.1	11.7	–

North-Central	459	7.2	6.1	–

Uva	452	7.1	6.1	–

Sabaragamuwa	625	9.9	9.7	–

**Household SES quintile**				

Poorest	1,539	24.3	20.1	20.0

Poorer	1,264	19.9	20.0	20.0

Middle	1,205	19.0	20.0	20.0

Richer	1,135	17.9	19.6	20.0

Richest	1,199	18.9	20.3	20.0

**Area SES tertile**				

Least developed	2,313	36.5	32.9	33.3

Middle	1,807	28.5	32.0	33.3

Most developed	2,222	35.0	35.1	33.3

**Body mass index (Mean)**	6,342	4.6 (17.2)	23.9 (4.6)	–

<25	3,928	62.4	61.0	–

25–29.9	1,794	28.5	29.3	–

30+	575	9.1	9.7	–

**Systolic BP (Mean ± SD)**	6,342	126.5 (19.6)	124.0 (18.5)	–

**Diastolic BP (Mean ± SD)**	6,342	79.6 (11.8)	78.7 (11.5)	–

**Previously diagnosed diabetic**	1,070	16.9	14.1	–

**Previously diagnosed hypertensive**	1,495	23.6	17.1	–

**Taking antihypertensive medication**	1,165	18.4	12.8	–


*Notes*: In the sector categorization, rural/estate refers to areas that were a mix of rural and estate sectors. Muslim ethnicity combines those who self-identified as Muslim or Malay. Population reference values taken from the 2012 national census statistics and adjusted for demographic change during 2012–2019.

### Hypertension prevalence

The overall weighted prevalence of hypertension among all Sri Lankan adults was 28.2% (95% CI: 26.6–29.7) using the JNC7 definition, which almost doubled to 51.3% (95% CI: 49.6–53.1) using the ACC/AHA 2017 definition. For adults aged ≤69 years, the corresponding estimates were 24.7% (95% CI: 23.0–26.3) and 48.9% (95% CI: 47.0–50.8), respectively. Of those classified as hypertensive (HTN-JNC7), 53.4% (weighted estimate) were previously diagnosed or on antihypertensive medication, and the remaining 46.6% were undiagnosed. Using the ACC/AHA 2017 definition, the corresponding ratios were 31.2% and 68.8%, respectively. Table 2 summarizes the prevalence estimates by major characteristics. When reweighting to the WHO standard population, prevalence for all adults was 26.6% (95% CI: 25.0–28.2) for JNC-7 and 49.8% (95% CI: 48.0–51.6) for ACC/AHA 2017.

**Table 2:** Prevalence of hypertension in Sri Lankan adults, SLHAS 2018–19.


CHARACTERISTICS	HYPERTENSION BY JNC7 % (95% CI)	HYPERTENSION BY ACC/AHA 2017 % (95% CI)	F-STATISTIC (P-VALUE)

**Overall**	28.2 (26.6–29.7)	51.3 (49.6–53.1)	

**Age (years)**			

18–29	6.7 (4.7–8.7)	29.8 (26.2–33.3)	104.7 (<0.001)

30–39	14.4 (11.8–17.0)	39.5 (35.9–43.1)	

40–49	27.2 (24.2–30.2)	55.4 (52.5–58.3)	

50–59	40.0 (36.8–43.2)	64.8 (61.3–68.2)	

60–69	56.4 (52.6–60.2)	74.0 (71.1–76.9)	

70–79	68.5 (64.6–72.4)	80.3 (77.2–83.4)	

80+	65.9 (58.0–73.8)	79.2 (72.0–86.3)	

**Sex**			

Male	28.1 (25.9–30.3)	51.8 (49.2–54.4)	0.0 (0.945)

Female	28.2 (26.4–30.0)	50.9 (48.7–53.2)	

**Ethnicity**			

Sinhala	27.1 (25.3–28.9)	50.0 (48.0–51.9)	2.32 (0.101)

Tamil	28.5 (24.9–32.2)	52.8 (47.4–58.1)	

Muslim	35.7 (29.9–41.5)	59.7 (54.2–65.2)	

Other	37.2 (10.0–64.5)	61.9 (37.0–86.8)	

**Education**			

No formal schooling	45.4 (36.2–54.7)	62.8 (52.9–72.6)	32.25 (<0.001)

Primary education	42.6 (38.3–46.8)	61.0 (56.3–65.8)	

Secondary education	26.1 (24.3–27.8)	50.0 (48.1–51.9)	

Tertiary education	23.6 (17.9–29.4)	47.2 (39.2–55.2)	

**Sector**			

Urban	45.4 (36.2–54.7)	62.8 (52.9–72.6)	1.83 (0.163)

Rural	42.6 (38.3–46.8)	61.0 (56.3–65.8)	

Estate	26.1 (24.3–27.8)	50.0 (48.1–51.9)	

Rural/estate	23.6 (17.9–29.4)	47.2 (39.2–55.2)	

**Household SES quintile**			

Poorest	27.2 (24.5–29.8)	48.6 (45.6–51.7)	1.25 (0.291)

Poorer	25.9 (22.9–28.9)	50.5 (47.1–53.9)	

Middle	28.6 (25.5–31.7)	51.3 (47.2–55.5)	

Richer	29.0 (26.1–32.0)	52.3 (48.7–56.0)	

Richest	30.1 (26.3–34.0)	54.0 (49.6–58.3)	

**Area SES tertile**			

Least developed	25.3 (22.9–27.6)	48.8 (45.9–51.7)	8.90 (0.003)

Middle	26.6 (23.9–29.3)	49.3 (46.0–52.5)	

Most developed	32.3 (29.5–35.1)	55.6 (52.6–58.7)	

**Body mass index (kg/m**^2^)			

<25	23.1 (21.5–24.7)	42.9 (40.8–45.0)	4.62 (0.033)

25–29.9	34.0 (31.5–36.5)	62.0 (59.3–64.7)	

30+	40.6 (34.9–46.2)	70.9 (66.2–75.6)	

**Previously diagnosed diabetic**	53.5 (49.4–57.6)	74.0 (70.4–77.6)	187.95 (<0.001)

**Previously diagnosed hypertensive**	88.2 (85.9–90.4)	93.7 (91.9–95.4)	874.45 (<0.001)


*Notes*: F-statistic for test of equal prevalence across groups is defined by each characteristic, computed for hypertension defined according to the JNC7 guidelines.

**Figure 1:**
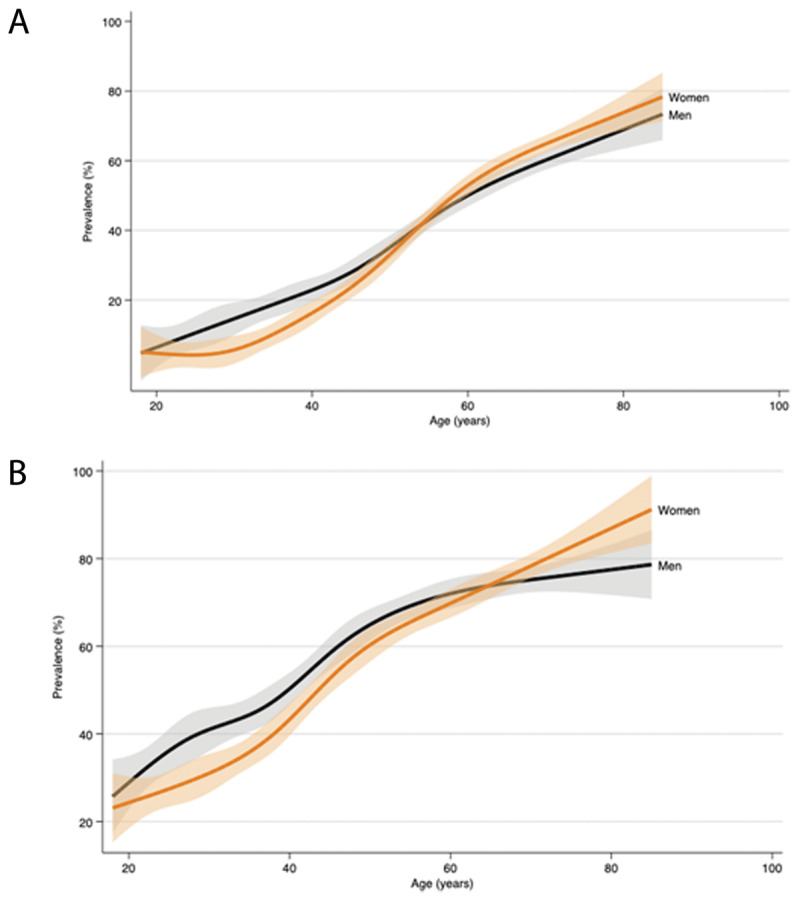
Age/sex-specific profiles of hypertension prevalence in Sri Lankan adults according to JNC7 and ACC/AHA 2017 definitions, SLHAS 2018–2019. *Notes*: Panel A displays the smoothed profile of HTN (JNC7) with age, and panel B displays the smoothed profile of HTN (ACC/AHA). Both are estimated using the weighted data for participants aged 18–85 years by fitting restricted cubic splines with six knots to allow for nonlinear relationships. Shaded regions denote 95% confidence intervals.

Hypertension prevalence varied similarly with both definitions across major characteristics. HTN-JNC7 prevalence increased almost linearly with age (1.4% per year) between 30 and 69 years, peaking in those aged 70–79 years (68.5%, 95% CI: 64.6–72.4), with evidence of plateauing and even a small fall in those aged ≥80 years. The increase in HTN-ACC/AHA with age was less steep, with the differences applying the two definitions being greatest in those aged 40–49 years, with prevalence doubling from 27.2% to 55.4%. With both definitions, overall prevalence was similar in both men (HTN-JNC7: 28.2%; HTN-ACC/AHA: 51.9%): and women (HTN-JNC7: 28.2%; HTN-ACC/AHA: 50.9%). However, the age-related increase was steeper in women from their 30s (Figure 1), with prevalence lower in women aged <40 years (HTN-JNC7: 7.8% vs. 12.6%) and higher in women aged ≥60 years (HTN-JNC7: 65.1% vs. 56.0%) than men of similar ages.

Across ethnic groups, prevalence was higher (*p* < 0.01) in Muslims, less educated adults, urban and rural locations, and Western and Central provinces, but these differences may be confounded by differences in age, ethnicity, and SES and require further investigation. HTN was highest in the richest SES quintile and the most developed area SES tertile. It was higher in those previously diagnosed as diabetic or taking diabetic medication, and prevalence also increased with BMI category, with 40.6% and 70.9% of those who were obese (BMI ≥30) being hypertensive according to the JNC7 and ACC/AHA 2017 definitions, respectively.

ACC/AHA 2017 guidelines recommend treatment with antihypertensive medication in individuals with Stage 1 HTN who have CVD or 10-year CVD risk >10%, whereas the WHO 2021 guidelines recommend medical therapy in those with diabetes or high CVD risk (undefined). It was beyond the scope of this analysis to comprehensively assess CVD status or to compute CVD risk, but the data yield a proxy measure that is history or medication consistent with CVD/PVD or prior diabetes diagnosis. Of the estimated 23.2% (95% CI: 22.1–24.5) of Sri Lankan adults classified as Stage 1 HTN, 16.6% (95% CI: 14.2–19.4) have a history or medication consistent with CVD/PVD or diabetes.

### Assessment of study design elements

Our final sample consisted of 139 urban and 159 nonurban PSUs, with average cluster sizes of 13.8 and 27.8, respectively. Estimation of ICCs confirmed spatial autocorrelation of HTN prevalence and BP at the PSU level: ICCs were 0.039 for hypertension (HTN-JNC7), 0.050 for SBP, and 0.075 for DBP. Spatial autocorrelation was also greater in urban (ICC = 0.039) than nonurban (ICC = 0.029) PSUs despite smaller cluster sizes and increased interhousehold intervals.

The expectation that hypertension would be correlated with area SES was confirmed. HTN was significantly higher (*p* < 0.001) in the most developed area SES tertile (Table 2), and regression analysis confirmed a strong association of HTN with area SES centile (*p* < 0.001 for both HTN measures), whereas localized regression revealed a linear relationship between the 3rd and 10^th^ decile of area SES, increasing from 32% to 45% (HTN-JNC7). When ethnicity was excluded from the weighting process, HTN prevalence fell to 27.8% (HTN-JNC7) and 51.1% (HTN-ACC/AHA), with DEFFs of 1.97 and 1.85, respectively, compared with our actual final DEFFs of 1.96 and 1.98, respectively.

## Discussion

This is the first study using a comprehensive national sample to estimate prevalence of HTN in all Sri Lankan adults, and it adds to the few studies assessing implications of the ACC/AHA 2017 definitions for HTN prevalence in LMICs.

We estimated that 28% (27% with age standardization) of Sri Lankan adults had hypertension (HTN-JNC7). These are higher than the NCD Risk Factor Collaboration estimates for Sri Lanka, indicating that hypertension is more prevalent than previous global estimates [1,26].

The ACC/AHA 2017 definition increases this by 23 percentage points to half the population (51.5%), similar to increases of 26 and 21 percentage points reported for Bangladesh and China, respectively [7,9], indicating that adopting the newer definition is likely to increase HTN prevalence by one in four adults in most Asian developing countries, or double overall prevalence. Of those reclassified as hypertensive, 17% had history or symptoms consistent with CVD/PVD or diabetes. As actual CVD and diabetes prevalence can be expected to be at least one and a half times more given likely rates of underdiagnosis [18], and because additional persons might be considered at high CVD risk, it is reasonable to infer that both ACC/AHA 2017 and WHO 2021 guidelines would recommend medical therapy in at least one in three to one in two Sri Lankans with Stage 1 HTN, increasing the number of adults recommended for treatment from under one-third to four in ten. Although this would increase health systems costs, this is likely to be cost saving in Sri Lanka by reducing morbidity, as has been found for China [7], but this warrants further analysis.

Our prevalence estimates (HTN-JNC7) are higher than some previous Sri Lankan studies that used subnational samples, but this could be explained by coverage differences and increasing prevalence over time. However, our estimate for adults aged <70 years (24.6%, 95% CI: 23.0–26.2) is consistent with the Sri Lanka STEPS 2015 study (26.1%, 95% CI: 24.4–27.2).

Our findings that HTN increases with urban residence, obesity, diabetes, and SES confirm patterns observed previously in Sri Lanka and other LMICs. The evident socioeconomic gradient in prevalence indicates that factors associated with greater affluence, such as obesity, salt intake, and exercise, play a major role in increasing risk. This suggests the need for greater focus on health education, screening, and treatment in urban and higher-income adults.

Compared with Bangladesh and India [8,9,27], the age-related increase in prevalence in Sri Lanka is steeper, with prevalence lower in younger adults (≤40 years) and higher in older adults (≥55 years). The increase is also steeper in women than in men from their 30s (Figure 1), and it closely matches the sex-specific life course trajectories of blood pressure in developed countries [28]. Observation of the same pattern in Sri Lanka adds evidence in favor of this being driven by physiological sexual dimorphism rather than environmental factors. This would imply that greater attention should be given to screening for hypertension and to managing blood pressure in older women, both in Sri Lanka and elsewhere.

Our study design incorporated several features to improve precision and reduce bias of estimates. The ICC estimates indicate significant spatial autocorrelation of HTN, consistent with a detailed Tanzanian study that reported a much higher ICC of 0.10 [29]. Our observed ICC of 0.039 is equivalent to a DEFF of 1.79, suggesting that the other design elements did not significantly increase the final DEFF and may have helped reduce it. This is consistent with the assumption of DEFFs of 1.5–2.0 in the design of many hypertension surveys, including the WHO STEPS and SAGE surveys where the assumption is not specifically for HTN but for all indicators [30,31]. However, when we reviewed recent national studies from South Asian and WHO South-East Asia Region countries, we found that although almost none reported their final obtained DEFFs, the mean DEFF derived from their prevalence estimates was 7.3 (median 3.6, range 1.5–22.2) (Supplementary Appendix). DEFFs also appear higher in other well-regarded surveys in developed countries. For example, prevalence estimates [32,33] using the United States NHANES data are associated with DEFFs >5. This suggests that researchers undertaking HTN prevalence surveys with complex designs would be safer to assume DEFFs of 3–5 in the absence of locally specific estimates and should consider additional design elements such as minimizing cluster sizes and using SES for additional stratification to increase precision. Because most researchers don’t report the actual DEFFs obtained, even if they report their assumptions in developing their design, we also suspect that most do not appreciate that DEFFs may often be much higher than 2. Consequently, we recommend other researchers to estimate and report the actual DEFFs obtained to increase awareness of these discrepancies and promote improvement of survey designs.

The inclusion of ethnicity in the weighting procedure eliminated a bias of 0.4% (HTN-JNC7), with trivial impact on DEFFs, validating the judgement that any bias reduction would outweigh loss of sample precision from the more complex weights calibration. This suggests that in populations where HTN varies substantially by ethnicity (or other characteristics for which population proportions are known), researchers should incorporate such characteristics in the weighting process if their samples are not representative along those dimensions.

### Strengths and limitations

The strengths of this study are that it used a large, nationally representative survey covering all demographic segments and districts in Sri Lanka; employed trained, dedicated field staff using standard procedures to take clinical measurements; undertook home examinations of respondents physically unable to attend a field clinic; and appropriately accounted for its complex survey design when making estimates. Poststratification weighting also ensured representativeness along multiple dimensions, and area SES stratification may have increased precision.

The overall SLHAS response rate of 65% is lower than reported by many health surveys in Sri Lanka. This can be explained by upweighting during recruitment of demographics anticipated to have lower response rates (e.g., young adults and men), disruption to survey operations caused by terrorist bombings, and rigorous operating procedures that prevented recruiters substituting another household member if the selected respondent refused, something that we have observed taking place in other Sri Lankan surveys. Nevertheless, our analysis specifically adjusted for nonresponse and observed biases, controlling for a wide range of factors, and our data were near complete for all participants, minimizing nonresponse bias.

A limitation of this analysis is that it cannot identity whether factors associated with higher prevalence are true risk factors. Further analysis using multivariate analysis that can simultaneously control for all covariates is required, and the authors plan to do this. In addition, the cross-sectional nature of the study further limits establishment of causality. However, the longitudinal nature of the SLHAS study does offer opportunities in future to identify what factors drive increases in hypertension. We also acknowledge that guidelines recommend measurement of blood pressure over more than one visit, which was not feasible given our study design and resources.

## Conclusion

We report the first nationally representative estimates of hypertension in Sri Lanka covering all adult ages, which can serve as a baseline for the SLHAS to track changes. Adopting the ACC/AHA 2017 definitions would almost double hypertension prevalence, but as many as one in three to one in two of those reclassified as hypertensive might have indications for medical therapy even under recent WHO guidelines. Our findings also add evidence in favor of a physiological basis for sexual dimorphism in prevalence.

Our study findings suggest that design effects of >5.0 should be considered more typical in hypertension surveys instead of the 1.5–2.0 commonly assumed, and reducing cluster sizes, increasing spatial distance between sampled households, and using area SES stratification might help improve precision.

## Data Accessibility Statement

Under the SLHAS Open Data policy, study data can be shared on reasonable request to the corresponding author after 2023.

## Additional File

The additional file for this article can be found as follows:

10.5334/gh.1135.s1 Supplementary Appendix. Analysis of design effects (DEFFs) in hypertension prevalence surveys in WHO South-East Asia region.
