# Supplementary material for: Prevalence and Associations of Hypertension in Sri Lankan Adults: Estimates from the SLHAS 2018–19 Survey Using JNC7 and ACC/AHA 2017 Guidelines
Source: Glob Heart. 2022 Aug 1;17(1):50. doi: 10.5334/gh.1135 (PMC9354554; doi:10.5334/gh.1135)
Supplement: Supplementary Appendix. — Analysis of design effects (DEFFs) in hypertension prevalence surveys in WHO South-East Asia region. [file gh-17-1-1135-s1.pdf]

Supplementary Appendix
Analysis of design effects (DEFFs) in hypertension prevalence surveys in WHO South-East Asia region

R.P. Rannan-Eliya, N. Wijemunige, P. Perera, Y. Kapuge, JRNA Gunawardana, C. Sigera, H.M.M. Herath, B. Perera, A. Gamage, N. Weerawardena, I. Sivagnanam, and SLHAS Collaborators. (2022)

General approach

We undertook a secondary analysis of recent hypertension prevalence surveys conducted in South Asia and the WHO South-East Asia region (SEAR) to assess design effects (DEFFs) in survey estimates. For each country, we identified the most recent survey used by the NCD Risk Factor Collaboration (NCD-RisC) in their 2021 estimates of global hypertension prevalence.[1] We then used the reported confidence intervals associated with their prevalence estimates to infer the study design effect.

Computation of design effects

The use of cluster sampling and complex survey designs reduces the precision of a survey compared with a survey that uses simple random sampling (SRS), owing to correlation between observations in the same cluster. The design effect is a measure of the impact of the impact of the survey design on precision, specifically the ratio of the variance associated with the survey estimate generated from that design to the variance associated with an estimate produced by a survey that used SRS.

When we reviewed the different country studies, we found that few prevalence studies that use complex survey designs report the actual design effects associated with their final prevalence estimates, although some do report the design effect assumed when setting the original sample target. However, if the study correctly computed the variances or confidence intervals associated with their point estimates, *i.e.*, appropriately accounted for the complex survey design during data analysis, then it is possible to infer the design effects from the reported confidence intervals.

Mathematically, the actual sample size divided by the design effect is the sample size that would be needed to achieve the same variance using a SRS design. Conveniently, this means that if the variance of an estimate generated by a complex survey design is known, then the design effect can be computed as the ratio of the actual sample size to the sample size required under SRS to achieve the same variance, since variance is given by the identity:

$$Variance, \sigma^2 = npq$$

where *n* is the sample size, *p* is the prevalence, and *q=(1-p).* For studies that report 95% confidence intervals (*95% CI*), the approximate variance can be derived using the standard relationship between the variance and the confidence interval, *i.e.*,

$$Variance, \sigma^2 = \left(\frac{95\%\ CI}{(1.96 \times 2)}\right)^2$$

Data

For each country, we considered the most recent survey reported by the NCD Risk Factor Collaboration (NCD-RisC) that reported either the design effect or the confidence intervals associated with the prevalence estimate.

None of the studies examined reported actual design effects, so the design effect was estimated in practice in all cases from the reported confidence intervals and sample sizes. For study reports that did not report an overall prevalence and confidence interval, the DEFF was computed as the mean of the design effects for the separate estimates for prevalence in men and women.

Results

Out of 13 countries considered, we located studies for all. Out of these, we could derive design effects for 12, of which 9 could be considered credible and are listed in Table 1. In three other studies, listed in Table 2, the inferred design effects were close to or less than 1 and there was no explicit explanation in the descriptions that the analysis had considered the complex survey design, despite all using a complex survey design with cluster sampling, suggesting that the investigators had not correctly accounted for the complex survey design when computing confidence intervals. One other study from Timor-Leste reported no confidence intervals and is also listed in Table 2.

For the 12 studies, where the implied values were considered credible, the mean design effect was 7.3, the median 3.6, and the range 1.5–22.2.

We additionally estimated the design effect for a recent study that estimated hypertension prevalence in the United States using US NHANES data. Details are given in Table 2.

Table 1: Estimated design effects in recent hypertension studies in South Asian and WHO SEAR countries, and United States

Country	Year	Survey type	Prevalence (95% CI)	Sample size	Estimated DEFF	Source
Afghanistan	2018	STEPS	29.2 (25.4–32.9)	3,956	7.0	[2]
Bhutan	2014	STEPS	35.7 (32.8–38.7)	2,912	2.9	[3]
India	2015–2016	DHS	18.1 (17.8–18.4)	731,864	11.6	[4]
Indonesia	2014–2015	LCS	33.4 (32.7–34.0)	29,965	1.5	[5]
Myanmar	2014	STEPS	26.4 (23.2–29.5)	8,757	11.6	[6]
Nepal	2019	STEPS	24.5 (22.4–26.7)	5,593	3.6	[7]
Pakistan	2014	STEPS	37.0 (34.9–39.0)	6,613	3.1	[8]
Sri Lanka	2014	STEPS	26.1 (24.4–27.7)	5,188	1.9	[9]
Thailand	2009	NHES	M: 41.5 (37.1–46.0)<br>F: 47.1 (42.1–52.1)	18,629	22.2[1]	[10]

Notes: [1]Mean of DEFFs for prevalence in men (M) and women (W). DHS= Demographic and Health Survey. LCS= Longitudinal cohort study. NHES=national health examination survey. STEPS= STEPS survey or survey using STEPS methodology.

Table 2: Recent hypertension studies in South Asian and WHO SEAR countries where confidence intervals assessed as not correctly computed, and United States

Country	Year	Survey type	Prevalence (95% CI)	Sample size	Implied DEFF	Source
Bangladesh	2017–2018	DHS	M: 24.3 (23.1–25.4)<br>F: 24.6 (23.6–25.6)	11,959	**1.0**[1]	[11]
Maldives	2011	STEPS	16.6	1,780	–	[12]
North Korea	2005	STEPS	M: 19.4 (18.5–20.3)<br>F:18.0(15.6–20.4)	2,125	0.6[1]	[13]
Timor-Leste	2014	STEPS	39.3	2,609	1.5	[14]
United States	2017–2018	NHES	31.7 (28.7–34.8)	4,730	5.3	[15]

Notes: [1]Mean of DEFFs for prevalence in men (M) and women (F). DHS= Demographic and Health Survey. NHES=national health examination survey. STEPS= STEPS survey or survey using STEPS methodology.

References

1. N. C. D. Risk Factor Collaboration. Worldwide trends in hypertension prevalence and progress in treatment and control from 1990 to 2019: a pooled analysis of 1201 population-representative studies with 104 million participants. Lancet. 2021;398(10304):957-80.

2. Ministry of Public Health. WHO STEPS noncommunicable disease risk factor surveillance in Afghanistan. Afghanistan 2018.

3. Sithey G, Wen LM, Dzed L, Li M. Noncommunicable diseases risk factors in Bhutan: A secondary analysis of data from Bhutan's nationwide STEPS survey 2014. PLoS One. 2021;16(9):e0257385.

4. Prenissl J, Manne-Goehler J, Jaacks LM, Prabhakaran D, Awasthi A, Bischops AC, et al. Hypertension screening, awareness, treatment, and control in India: A nationally representative cross-sectional study among individuals aged 15 to 49 years. PLoS Med. 2019;16(5):e1002801.

5. Peltzer K, Pengpid S. The Prevalence and Social Determinants of Hypertension among Adults in Indonesia: A Cross-Sectional Population-Based National Survey. Int J Hypertens. 2018;2018:5610725.

6. Department of Public Health, Department of Medical Research. Report on National Survey of Diabetes Mellitus and Risk Factors for Non-communicable Disease in Myanmar. 2014.

7. Bista B, Dhimal M, Bhattarai S, Neupane T, Xu YY, Pandey AR, et al. Prevalence of non-communicable diseases risk factors and their determinants: Results from STEPS survey 2019, Nepal. PLoS One. 2021;16(7):e0253605.

8. Rafique I, Saqib MAN, Munir MA, Qureshi H, Rizwanullah, Khan SA, et al. Prevalence of risk factors for noncommunicable diseases in adults: key findings from the Pakistan STEPS survey. Eastern Mediterranean Health Journal. 2018;24(01):33-41.

9. Ministry of Health, Nutrition and Indigenous Medicine (MoHNIM). Non Communicable Disease Risk Factor Survey Sri Lanka 2015. Colombo: MoHNIM; 2015.

10. Aekplakorn W, Chariyalertsak S, Kessomboon P, Sangthong R, Inthawong R, Putwatana P, et al. Prevalence and management of diabetes and metabolic risk factors in Thai adults: the Thai National Health Examination Survey IV, 2009. Diabetes Care. 2011;34(9):1980-5.

11. Rahman MA, Halder HR, Yadav UN, Mistry SK. Prevalence of and factors associated with hypertension according to JNC 7 and ACC/AHA 2017 guidelines in Bangladesh. Sci Rep. 2021;11(1):15420.

12. World Health Organization, Health Protection Agency (Maldives). WHO STEPS survey on risk factors for noncommunicable diseases Maldives, 2011.2011.

13. World Health Organization, Ministry of Public Health. Final Report of Non-Communicable Disease (NCD) risk factor survey in Moranbong District of Pyongyang DPR Korea. DPR Korea: WHO, MoPH; 2006.

14. World Health Organization. National survey for noncommunicable disease risk factors and injuries using WHO STEPS approach in Timor-Leste. Timor-Leste: WHO; 2014.

15. Rana J, Oldroyd J, Islam MM, Tarazona-Meza CE, Islam RM. Prevalence of hypertension and controlled hypertension among United States adults: Evidence from NHANES 2017-18 survey. Int J Cardiol Hypertens. 2020;7:100061.
